# From mindfulness to work engagement: The mediating roles of work meaningfulness, emotion regulation, and job competence

**DOI:** 10.3389/fpsyg.2022.997638

**Published:** 2022-10-26

**Authors:** Liang Chen, Xiaobei Li, Lu Xing

**Affiliations:** ^1^Department of Business and Administration, East China University of Science and Technology, Shanghai, China; ^2^Department of Business Administration, Shanghai Business School, Shanghai, China; ^3^Department of Business Administration, Hunan University, Changsha, Hunan, China

**Keywords:** mindfulness, work meaningfulness, emotion regulation, job competence, work engagement

## Abstract

Drawing from the grounded theory of work engagement, this research aims to explore three essential yet previously unexamined pathways—work meaningfulness, emotion regulation, and job competence in simultaneously transmitting the effects of mindfulness training to employee experience of work engagement. We employed a six-wave quasi-experimental design and recruited 129 employees (77 from experimental group and 59 from control group) to participate in the quasi-experiment, and tested our simultaneous mediating models using the structural equation modeling. Results showed that mindfulness facilitated employees’ work meaningfulness, emotion regulation, and job competence, which in turn enhanced employee work engagement. By doing so, we add to the mindfulness literature by showing that the three essential psychological states are important machanims that link mindfulness to work engagement. Practicially, this research reveals that mindfulness training is an effective tool to influence employees’ psychological states (e.g., meaningfulness, competence), which ultimately develop their work engagement in the workplace.

## Introduction

Engaged employees not only focus their physical effort on the tasks at hand, but are also cognitively vigilant and emotionally connected to the endeavor (Kahn, 1990; Ashforth and Humphrey, 1995). A recent investigation shows that every one percent growth in employee engagement corresponds with 57–68 million dollars increase in company operating profit (SAP, 2020). Similarly, Rich et al. (2010) have convincingly demonstrated that highly engaged employees devoted their physical, cognitive, and emotional resources to solving work-related issues, which lead to favorable organizational outcomes including higher productivity and customer satisfaction, but fewer mistakes or accidents, as compared to their less engaged peers (Harter et al., 2002; Christian et al., 2014).

Referring to a positive and fulfilling state of mind in one’s work role, work engagement indicates employees’ full of energy, absorption, and dedication to their job (Schaufeli et al., 2002). Given the beneficial roles of work engagement, a critical question that attracts both managers and scholars is: how can work engagement be developed? Recent studies on mindfulness (Hafenbrack et al., 2020; Miyahara et al., 2022; Shaffakat et al., 2022) suggest that mindfulness as a state of consciousness (Brown et al., 2007) is one potential answer. More specifically, state mindfulness is characterized by sustained attention to, clear awareness of, and acceptance of, present events and experiences (Brown et al., 2007; Glomb et al., 2011). It enhances individuals’ attention regulation through a process called “decentering” where individuals attend to moment-to-moment experiences by simply observing the internal and external stimuli without evaluation or judgment, and therefore creates distance between the self and the experience (Brown et al., 2007; Fresco et al., 2007). In the last decade, several studies have shown the connection between mindfulness and engagement from different theoretical perspectives (for a review, please see Shahbaz and Parker, 2021). For example, using self-determination theory, Leroy et al. (2013) argued that the inner awareness due to high mindfulness can facilitate individuals to build up high levels of authenticity function, and finally increases individuals’ work engagement. Moreover, based on the psychological capital theory and with a sample of 299 adults in full-time employment, Malinowski and Lim (2015) demonstrated that self-reported mindfulness, as an individual’s ability to step back from automatic, habitual reactions to distress, exerts its positive effect on work engagement by increasing positive affect, hope, and optimism. More recently, drawing on the theory of conservation of resources, Liu et al. (2020) argued that high mindfulness enables individuals to avoid expending psychological energy, save current recovery level and experience greater autonomy and control. Based on the analysis of data from 311 employees at different times, they showed that mindfulness has a positive influence on work engagement through the individual’s recovery level. Practically, in recent years many companies such as Google, Didi, Facebook, and Apple have adopted mindfulness training to foster employee engagement (Hochman, 2013; Schaufenbuet, 2015).

Building on these findings on the relationship between mindfulness and work engagement, in this paper we aim to further explore the potential underlying pathways by returning to the roots of work engagement theory—Kahn (1990). According to Kahn (1990), three psychological states—meaningfulness, safety, and availability are the preconditions for workplace engagement. More specifically, meaningfulness describes a feeling of worthiness, usefulness, and valuableness (Kahn, 1990). When applied to the workplace, work meaningfulness refers to the feeling of work being valuable, judged by individuals’ own ideas or standards (e.g., Thomas and Velthouse, 1990; Spreitzer, 1995). Safety, according to Kahn (1990), is a feeling of being able to show oneself without fear of receiving negative consequences regarding self-image. In a work setting, such state can be reflected by an individual’s emotion regulation in which negative emotions such as fear and nervous are aware of, accepted, and their associated impulsive behaviors are effectively controlled for. Finally, with high psychological availability, individuals experience readiness both physically and mentally for role performance, which indicates a sense of competence people have regarding their capability in completing their tasks with necessary skills and resources (Gist, 1987). Accordingly, in this study, we attempt to explore meaningfulness, emotion regulation, and job competency as three psychological mechanisms that connect mindfulness to work engagement.

More specifically, mindfulness helps bring positive change in employees’ work meaningfulness because mindfulness fulfills individuals’ essential needs such as transcendence (Kirkpatrick and Locke, 1996; Breen, 2007), based on which employees are more likely to perceive the bigger purpose of their jobs and consider their job as meaningful. Mindfulness also enables employees to be aware of and accept their negative emotions and redirect their emotions to goal-oriented tasks (Brown and Ryan, 2003; Brown et al., 2007). In this way, individual negative emotions such as fear are effectively regulated (Brown and Ryan, 2003; Brown et al., 2007), cultivating a state of safety and emotional readiness for work engagement. Finally, mindfulness reduces mind wandering amid competing demands and help individuals stay focused on their present tasks (Ocasio, 2011), thus elevating their perceived job competence. Taken together, we posit that mindfulness facilitates employees’ perceived work meaningfulness, emotion regulation, and job competence, and in turn enhance their work engagement.

As such, our paper aims to contribute to mindfulness and engagement literature by identifying work meaningfulness, emotion regulation, and job competence as three foundamental yet ignored pathways to connect mindfulness with work engagement. As noted earlier, previous scholars have shown that mindfulness reinforces employee engagement from different theoretical perspectives; however, according to the work engagement theory (Kahn, 1990), work engagement has its own implied mechanisms—meaningfulness, safety, and availability. Referring to this theory directly, we are able to understand how work engagement is achieved in a more fundamental way. In doing so, we also answered the calls of Allen and Kiburz (2012) and Dane (2011) to investigate mindfulness in the workplace and unfolds how it affects workplace engagement. Our focus on the roots of work engagement theory provides solid support to this theory and reinforces the far-reaching impacts of mindfulness at work. Our study therefore points to mindfulness training as a feasible way for employees’ improvement of work engagement.

## Hypotheses development

### Mindfulness and work meaningfulness

Because meaningful work is purposeful and significant (Pratt and Ashforth, 2003), the extent to which work can meet one’s essential needs will influence one’s experience of work meaningfulness (Michaelson et al., 2014). Mindfulness can enhance employees’ experience of work meaningfulness through fulfilling their two essential needs: flourishing (Breen, 2007) and transcendence (Kirkpatrick and Locke, 1996; Piccolo and Colquitt, 2006).

Flourishing refers to the realization of one’s potential or achieving effective functioning in life (Breen, 2007). One of the conditions identified to be critical for human flourishing and functioning is authenticity (e.g., Brown et al., 2007; Allen et al., 2015). Scholars have articulated how mindfulness can positively induce authenticity (e.g., Leroy et al., 2013). For example, Ilies et al. (2005) suggested that as mindfulness-induced self-awareness and self-acceptance increased, people would become more open to express their true selves. Similarly, Kernis and Goldman (2006) reasoned that the non-judgmental and receptive awareness accompanied with mindfulness mitigated egocentric regulation, and in turn led to integrated and authentic functioning. These arguments suggest that mindful employees are likely to experience work meaningfulness.

Meaningfulness can also be achieved through fulfilling the need for transcendence, that is, becoming part of something greater than self (Kirkpatrick and Locke, 1996; Piccolo and Colquitt, 2006). Research has shown that mindfulness practices tend to help transform people’s view of their relationships with the outside world, viewing the world in a more objective manner, increasing other-orientation, and decreasing ego-involved processing (Good et al., 2016). In the organizational setting, it means that mindful employees are more likely to feel to be part of the organization and develop a sense of how their work is related to others such as coworkers, the work team, customers, or the society. We thus propose:

*Hypothesis 1.* Mindfulness is positively related to employees’ experience of work meaningfulness.

### Mindfulness and emotion regulation

Mindfulness can also improve emotion regulation in the workplace (e.g., Arch and Craske, 2006; Hill and Updegraff, 2012; Hülsheger et al., 2013). Specifically, we focus on a integrative conceptualization of emotion regulation that involves not just the awareness, understanding, and acceptance of emotions, but also the ability to act in consistent with individuals goals (Gratz and Roemer, 2004). Building on previous studies arguing specific emotion regulation strategies (e.g., deep acting and surface acting) as critical mechanisms in the functioning of mindfulness (e.g., Hülsheger et al., 2013; Liang et al., 2018), we consider that mindfulness may enhance individual emotion regulation from a more integrative point of view, which contains “(a) awareness and understanding of emotions, (b) acceptance of emotions, (c) ability to control impulsive behaviors and behave in accordance with desired goals when experiencing negative emotions, and (d) ability to use situationally appropriate emotion regulation strategies flexibly to modulate emotional responses as desired in order to meet individual goals and situational demands (Gratz and Roemer, 2004, pp. 42–43)”.

Furthermore, as a multidimensional construct, mindfulness can reinforce individual emotion regulation in the following ways. First, in a mindful state, employees demonstrate high awareness of their inner feelings such as fear and their physical symptoms such as trembling and shortness of breath (Brown et al., 2007). Their complete attention to such emotional experiences thus opens the gate of their awareness and understandings of emotions, as well as distinguishing the subtle differences among distinct emotions (Brown and Ryan, 2003). As such, their perceptions of emotions are relatively clear and accurate (Brown and Ryan, 2003). Second, high mindfulness usually brings a nonjudgmental or accepting view regarding their own experiences (Kabat-Zinn, 1990; Brown and Ryan, 2003; Brown et al., 2013). Thus, when experiencing negative emotions, such as fear and anger, they are more inclined to accept their emotions as it is without falling prey to autonomous judgments such as associating their fear with individual weakness or inadequacy. Further, such acceptance also inhibits individual spontaneous reaction driven by their feelings (Kabat-Zinn, 1990; Brown and Ryan, 2003). Instead of rashly responding to felt fear with impulsive behaviors, a mindful employee tranquilly embraces his or her feelings, thus disenabling their impacts on subsequent behaviors. Third, mindfulness offers individuals with available cognitive resources for emotion regulation such that mindful employees are more capable of redirecting their focus and energies to their assignments at hand, so as to cope with goal demands (Grover et al., 2017). Taken together, instead of simply suppressing individual fear by surface acting (Hülsheger et al., 2013), mindful individuals pay attention to their ongoing experiences of negative emotions (e.g., fear), understand, accept, and embrace them in such a way that negative emotions are effectively resolved and regulated. Thus, we propose:

*Hypothesis 2. Mindfulness is positively related to employees’ emotion regulation.*

### Mindfulness and job competence

We propose job competence as an individual belief regarding their capacity in successfully completing assigned tasks. While numerous factors can influence one’s sense of job competence, we argue that mindfulness is the one that is under-studied. Mindful employees are more likely to demonstrate high job competency firstly due to their focus and wide attention to the present moment. Previous studies have elaborated that mindfulness faciliatates flexible and wide attention and thus boosts individual devotion to their tasks at hand and avoids distractions amid competing demands (e.g., Dane, 2011; Ocasio, 2011). As such, mindful employees are capable of completing their work assignments effectively (Brown et al., 2007), during which, a great sense of job competence may be developed.

Moreover, mindfulness may also enhance a sense of job competence through the state of non-judgmental and receptive awareness it creates. Mindful employees are likely to first be aware of task-related problems and identify which aspects need improvement for task accomplishment (Brown et al., 2007). Additionally, high awareness and non-jugmental view also mirror the actual situations (Brown and Ryan, 2003) via which their decision makings and goal settings are based on their accurate understandings of the reality. Consequently, mindful employees are more likely to come up with effective problem solving strategies tailored to the current situations (Shapiro et al., 2012). Additionally, high acceptance of the reality without judgment helps employees detach their self images from their work (Brown et al., 2007; Charoensukmongkol, 2017; Kroon et al., 2017). As such, they are more likely to persistent in trying new ways of accomplishing tasks without being obstructed by their negative self judgments (Kabat-Zinn, 1990) when receiving negative feedback. More importantly, being non-judgmental can free up mental resources that increase working memory capacity (e.g., Jha et al., 2010) and cognitive flexibility (Moore and Malinowski, 2009). It is thus likely that more mindful employees are equipped with more cognitive resources and flexibility that help them develop more insights and readiness to problem solving and undertake tasks more efficiently (Ostafin and Kassman, 2012). Accordingly, we propose:

*Hypothesis 3: Mindfulness is positively related to employees’ sense of job competence.*

### Work meaningfulness, emotion regulation, and job competence simultaneously mediate the relationship between mindfulness and work engagement

Building on Kahn’s grounded theory of work engagement, we propose work meaningfulness, emotion regulation, and job competence as three critical psychological conditions for employees to experience work engagement. More specifically, Kahn (1990) found—from his qualitative studies of summer camp counselors and construction workers—that those who felt more psychological meaningfulness were more willing to put their whole selves, i.e., their full energies into work. On the contrary, failing to perceive meaning in one’s work would lead to “disengagement” from work—when people withdraw from work and defend themselves physically, cognitively, and emotionally during role performance (Kahn, 1990; Aktouf, 1992).

In addition, research has shown that employees who experience work meaningfulness tend to believe that their work is vital in life, and thus are willing to put great efforts to it (Steger et al., 2012). Chalofsky and Krishna (2009) viewed meaningfulness as a deeper level of intrinsic motivation that explained employees’ work engagement. In a similar vein, scholars such as May et al. (2004) found that employees who believed that their work was personally meaningful were fully engaged, as meaningfulness brought positive affective and cognitive energy to their work (Fenton-O’Creevy et al., 2011). As such, meaningfulness was a strong predictor for work engagement, even beyond other factors such as job feedback (Christian et al., 2014). Following along previous studies, we propose that mindfulness reinforces employees’ work meaningfulness, which in turn promotes their work engagement. Hence, we propose:

*Hypothesis 4a.* Work meaningfulness mediates the positive relationship between employee mindfulness and work engagement.

Emotion regulation also acts a pathway via which employee mindfulness may facilitate work engagement. For those mindful employees who regulate their emotions well, they demonstrate high clarity regarding the emotions they experience and display high control on the extent to which their emotions drive specific behaviors (Hill and Updegraff, 2012; Liang et al., 2018). They are also capable of directing their emotional energies to goal-oriented activities (Hülsheger et al., 2013; Raphiphatthana et al., 2018), in this case, their work assignments. To them, emotion regulation cultivates an inner safe environment where emotions are utilized as weapons for work engagement, rather than as obstacles that prevent them from focusing on the work itself (Fenton-O’Creevy et al., 2011). As such, mindful employees feel free and safe to be devoted to work via emotion regulation, which cultivates emotional readiness for work engagement.

Additionally, emotion regulation has been shown to be negatively related to emotional exhaustion and facilitate recovery from negative emotions at work (Hülsheger et al., 2013). From this perspective, an employee’s emotion regulation reflects their capabilities to understand their emotions, to enhance impulse control, and to instill them with high levels of energy to handle work demands (Gratz and Roemer, 2004). In doing so, emotion regulation creates the preconditions for safe and positive working experiences, from which, work engagement is fostered. We therefore propose:

*Hypothesis 4b.* Emotion regulation mediates the positive relationship between employee mindfulness and work engagement.

We further argue that job competence will transit the positive impact of mindfulness to employees’ work engagement. Job competence helps sustain attention and energies at work because competent employees tend to do their jobs more efficiently, and subsequently receive more positive feedback and experience joy and satisfaction from work (Deci, 1975; Lepper and Greene, 1978; Bandura, 1981). Employees with high job competence also show high confidence and perseverance at work despite obstacles they may face (Raphiphatthana et al., 2018). Thus, they are more likely to become absorbed at work and demonstrate high persistence in attaining objectives. Such readiness also reinforces their motivation to gain positive influence at work and propels them to reach individual goals with confidence, and adequate skills and knowledge (Gist, 1987). Overall, employees with high job competence demonstrate psychological and mental readiness so as to be fully engaged at work (Kahn, 1990).

As support of our arguments, Maslach et al. (2001) identified a strong correlation between competence and engagement; Lau and Roeser (2002) found that students who perceived higher competence were more likely to committed to science in the form of situational engagement. Building on these findings, we further argue that job competence equips employees with necessary skills and resources (Gist, 1987), that foster their experienced vitality (Allen and Kiburz, 2012) and therefore enable them to be fully engaged for task accomplishments. As such, we propose:

*Hypothesis 4c.* Job competence mediates the positive relationship between employee mindfulness and work engagement.

## Materials and methods

### Design and participants

Following previous mindfulness studies (Hülsheger et al., 2013; Kay and Skarlicki, 2020), we conducted a quasi-experiment to test our hypotheses. In this quasi-experiment, 77 part-time MBA students with full employment in China participated in an 8-week mindfulness training, whereas 52 part-time MBA students who did not attend the mindfulness classes served as a control group. The control group were recruited through personal social network, with a snowball sampling approach (Vine, 2011). The experimental design therefore was 2 (mindfulness training: yes or no) × 2 (pre-intervention and post-intervention, repeated within-subjects).

In order to reduce the common method variance, and reduce respondents’ fatigue (Lance, 2010), we collected our pre-intervention data at three points in time with one week in between before the beginning of the mindfulness class. More specifically, at Time 1, participants rated their mindfulness levels; at Time 2, we asked questions on work meaningfulness, emotion regulation, and job competence; at Time 3, participants reported their work engagement levels. After eight weeks’ training, we continued to collect post-intervention questionnaires in Week 9 (Time 4) on mindfulness state, Week 10 (Time 5) on work meaningfulness, emotion regulation, and job competence, and Week 11 (Time 6) on work engagement. We sent out the survey to the control group and the experiment group at the same time, and then collected their responses again after the 8-week intervention.

For the experiment group, in total, 128 participants completed Time 1 questionnaire; 112 participants completed Time 2 questionnaire (87.50%), and 97 participants filled out Time 3 survey (75.78%). After the 8-week class, 82 participants completed Time 4 questionnaire (64.06%), 79 completed Time 5 questionnaire (61.72%), and finally 77 filled out Time 6 survey (60.16%). Overall, our final experimental group sample consisted of 77 employees. One key reason why the participants dropped out is that they could not attend every class due to business arrangements such as business trips. We conducted univariate ANOVA tests and did not find significant difference on gender and age between the dropouts and the remaining participants; neither did we identify significant difference on gender and age between participants in the experimental and control groups.

### Experimental treatment: Mindfulness training class

One author offered the eight-week mindfulness training classes for all participants in the experimental group. The class was conducted once every week and each class lasted for 3 h. The class employed Mindfulness-Based Stress Reduction (MBSR; Kabat-Zinn, 1990) as mindfulness training including practices such as mindful sitting meditation, body scan, and mindful movement. In addition to the class time, participants were required to practice these techniques for at least 20 min every day.

Regarding the control group, similar to other mindfulness experiment research (e.g., Michel et al., 2014; Althammer et al., 2021), we did not provide any intervention to the participants during the 8-week mindfulness training period.

### Measures

We adopted well-established measures that have shown high reliabilities and validities in previous research. All our surveys were administered in Chinese. We followed Brislin’s (1980) translation/back-translation procedures in translating all our measures from English into Chinese.

*State mindfulness* was measured using the Five Factor Mindfulness Questionnaire (Baer et al., 2006). All the 39 items were rated on a five-point Likert scale (1 = “rarely”; 5 = “almost always”). Sample items were “I perceive my feelings and emotions without having to react to them.” The alpha reliability for this scale was 0.88 and 0.90 for pre- and post-intervention, respectively.

*Work meaningfulness* was measured using a subscale of Organizational Commitment Questionnaire validated by Caldwell et al. (1990). The 3 items were rated on a five-point Likert scale (1 = “strongly disagree”; 5 = “strongly agree”). A sample item was “If the values of this organization were different, I would not be as attached to this organization.” The alpha reliability for this scale was 0.83 and 0.90 for pre- and post-intervention, respectively.

*Emotion regulation* was measured using the 11 items developed by Gratz and Roemer (2004). All the items were rated on a five-point Likert scale (1 = “strongly disagree”; 5 = “strongly agree”). A sample item was “I know exactly how I am feeling.” The alpha reliability for this scale was 0.79 for pre-intervention and 0.84 for post-intervention.

*Job competence*. We measured job competence using the 4-item scale developed by Yperen and Hagedoorn (2003) on a seven-point Likert scale (1 = “strongly disagree”; 7 = “strongly agree”). A sample item was “I do this job for the satisfaction I feel while overcoming certain difficulties in my job.” The reliability (Cronbach alpha) for this scale was 0.90 and 0.88 for pre- and post-intervention.

*Work engagement* was measured using Rich’s Job Engagement Scales (Rich et al., 2010). The 16-item scale measured physical engagement, emotional engagement, and cognitive engagement on a five-point Likert scale (1 = “strongly disagree”; 5 = “strongly agree”). Participants responded to items such as “I devote a lot of energy to my job.” The reliability (Cronbach alpha) for this scale was 0.91 and 0.92 for pre- and post-intervention.

*Control variables.* To exclude possible impacts from job and task contexts on our proposed relationships, we controlled for employees’ job demands and job resources, along with other demographic variables (i.e., age, gender, and marital status). We adopted the Job Demands and Resources Scale (Jackson and Rothmann, 2005) using a five-point Likert scale (1 = “never”; 5 = “always”) to measure job demands (α = 0.91) and job resources (α = 0.83). Participants’ ratings were collected in the Time 2 survey before the intervention with the mediating variables(work meaningfulness, emotion regulation, and job competence). We also controlled for pre-intervention ratings of the respective dependent variables, including work meaningfulness, emotion regulation, job competence, and work engagement to exclude their potential influences on that after the intervention.

### Analytic strategies

Repeated-measures univariate analyses of variance (RM ANOVA) was employed for the manipulation check of mindfulness. Confirmatory factor analysis was conducted to examine the discriminant validity of focal variables. The hypothesized model was tested in Mplus Version 7. Rather than using piecemeal or causal steps approaches, we performed path analysis to test our hypotheses simultaneously. All bias-corrected bootstrapping was implemented by drawing 2,000 random samples with replacement from the full sample.

## Results

### Manipulation check

We first checked the baseline levels of all variables (pre-intervention) between the experimental groups (*N_1_* = 77) and the control group (*N_2_* = 52) using univariate ANOVA. Results indicated that there were no significant differences in mindfulness (*F*_*N1* = 77,N2 = 52_ = 1.63, *p* = 0.19), job competence (*F*_*N1* = 77,N2 = 52_ = 0.54, *p* = 0.66), work meaningfulness (*F*_*N1* = 77,N2 = 52_ = 1.50, *p* = 0.22), emotion regulation (*F*_*N1* = 77,N2 = 52_ = 0.58, *p* = 0.63), or work engagement (*F*_*N1* = 77,N2 = 52_ = 2.13, *p* = 0.10).

We then conducted RM ANOVA to account for differences in baseline mindfulness and changes in mindfulness over time. The data met the sphericity assumption (i.e., equality of variances of between level differences) for within-group ANOVA (*Box’s M* = 22.12, *F* = 2.35, *p* = 0.01). Our results showed significant time by condition interactions for mindfulness (*F* = 13.29, *p* < 0.00, partial *η^2^* = 0.25, *alpha* = 1.00), indicating that mindfulness state increased significantly in the experimental condition, but not in the control condition. These results suggested that our manipulation of mindfulness was effective.

### Confirmatory factor analysis

We conducted confirmatory factor analysis to examine the discriminant validity of all of our variables, including mindfulness, work meaningfulness, emotion regulation, job competence, and work engagement. Our results demonstrated that our hypothesized five-factor model fit our data better [χ^2^(152) = 276.57, χ^2^/*df* = 1.82, SRMR = 0.06, RMSEA = 0.06, CFI = 0.94, TLI = 0.93] than alternative models. These results therefore provided support for the discriminant validity of our constructs.

### Hypotheses testing

**FIGURE 1:**
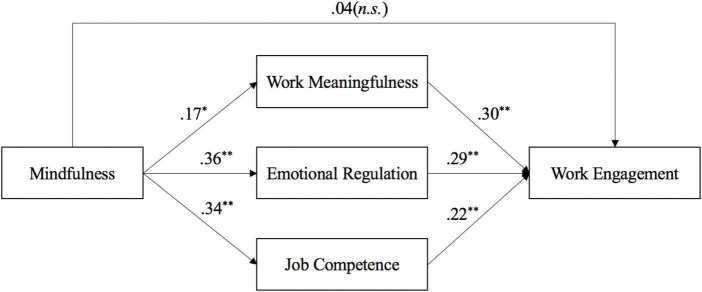
Hypothesized theoretical model and results. *Using mplus 7.0, N = 129.* All of the reported paths are standardized coefficients. Control variables are job demands and job resources rated at Time 1, demographic variables including age, gender and marital status, as well as the respective variables rated before our intervention. *p < 0.05; **p < 0.01.

Table 1 presents descriptive statistics and correlations among all variables. Hypothesis 1 stated that mindfulness was positively related to employee perceived work meaningfulness. As shown in Table 2 and Figure 1, there was a significant effect of mindfulness on work meaningfulness (β = 0.34, *p* < 0.05, 95% CI [0.001, 0.624]). These results supported Hypothesis 1.

**TABLE 1:** Means, standard deviations, and correlations^a^.

Variables	*M*	*SD*	1	2	3	4	5	6	7	8	9	10	11	12	13	14
1. Age	33.32	4.50														
2. Gender^b^	0.53	0.50	0.01													
3. Marriage status^c^	0.76	0.43	0.47**	0.06												
4. Job demands	2.86	0.65	0.28**	0.17	0.23**											
5. Job resources	3.54	0.69	0.12	–0.10	–0.04	0.26**										
6. Mindfulness Pr^d^	3.34	0.50	0.11	0.03	–0.04	–0.04	0.37**									
7. Mindfulness Po	3.59	0.49	0.00	–0.04	–0.08	–0.01	0.38**	0.53**								
8. Work meaningfulness Pr	3.02	1.01	0.01	–0.10	–0.03	0.12	0.41**	0.29**	0.34**							
9. Work meaningfulness Po	3.32	0.98	0.12	−0.20*	0.10	0.07	0.31**	0.16	0.36**	0.65**						
10. Emotion regulation Pr	3.88	0.50	0.10	–0.05	0.07	–0.11	0.17	0.29**	0.46**	0.20*	0.27**					
11. Emotion regulation Po	3.99	0.51	0.07	0.00	0.05	–0.05	0.11	0.19*	0.48**	0.08	0.21*	0.50**				
12. Job competence Pr	4.52	0.63	0.07	–0.02	0.04	0.11	0.35**	0.03	0.08	0.20*	0.07	–0.03	0.04			
13. Job competence Po	4.51	0.61	0.06	–0.08	–0.04	0.01	0.35**	0.15	0.42**	0.23**	0.30**	0.09	0.15	0.36**		
14. Work engagement Pr	3.73	0.63	0.14	–0.09	0.11	0.26**	0.55**	0.43**	0.45**	0.44**	0.35**	0.16	0.06	0.35**	0.39**	
15. Work engagement Po	3.85	0.65	0.15	–0.05	0.06	0.23**	0.39**	0.21*	0.45**	0.31**	0.46**	0.15	0.35**	0.36**	0.53**	0.64**

^a^*n* = 129, *n* = 77 for mindfulness intervention condition and *n* = 52 for control condition analysis.

^b^Male = 1; Female = 0.

^c^Married = 1; Not married = 0.

^d^Pr = Preintervention; Po = Postintervention.

**p < 0.01; *p < 0.05.

**TABLE 2:** Path analysis results.

Hypotheses		Unstandardized results	Confidence interval (95%)		Standardized results	Test result
H1	Mindfulness→Work meaningfulness	0.338*	0.001	0.624	0.167*	Supported
H2	Mindfulness→Emotion regulation	0.369**	0.216	0.556	0.355**	Supported
H3	Mindfulness→Job competence	0.429**	0.277	0.579	0.343**	Supported
	Work meaningfulness→Work engagement	0.201**	0.082	0.312	0.304**	
	Emotion regulation→Work engagement	0.377**	0.110	0.661	0.293**	
	Job competence→Work engagement	0.233**	0.083	0.414	0.218**	
	***Mediating effects***					
H4a	Mindfulness→Work meaningfulness→Work engagement	0.068*	0.005	0.164	0.051*	Supported
H4b	Mindfulness→Emotion regulation→Work engagement	0.139**	0.051	0.291	0.104**	Supported
H4c	Mindfulness→Job competence→Work engagement	0.100**	0.035	0.217	0.074**	Supported

*p < 0.05; **p < 0.01.

Hypothesis 2 predicted that mindfulness was also positively related to employee emotion regulation. Similarly, there was a significant effect of mindfulness on emotion regulation (β = 0.37, *p* < 0.01, 95% CI [0.22, 0.56]), providing supports for Hypothesis 2.

Hypothesis 3 predicted that mindfulness would be positively related to employees’ sense of job competence. As shown in Table 2 and Figure 1, there was a significant effect of mindfulness on job competence (β = 0.43, *p* < 0.01, 95% CI [0.28, 0.58]). These results provided support for Hypothesis 3. Besides, we calculated the propotion of variance in work engagement that can be explained by the three mediators. *R*^2^*-*change results showed that work meaningfulness, emotion regulation, and job competence repsectively explained 6, 6, and 3% of the variance of work engagement. That is, work meaningfulness and emotion regulation provide similar explained variance of work engagement. The contribution of each of these two is slightly larger than job competence.

Hypothesis 4a–4c stated that work meaningfulness, emotion regulation, and job competence simultaneously mediate the relationship between mindfulness training and work engagement. Results in Table 2 showed significant indirect effects from mindfulness training condition via work meaningfulness to work engagement (0.07, 95% CI [0.01, 0.16]), through emotion regulation to work engagement (0.14, 95% CI [0.05, 0.29]), and via job competence to work engagement (0.10, 95% CI [0.04, 0.22]). These results therefore offered strong support for our Hypothesis 4a, 4b, and 4c.

## Discussion

Returning to the roots of work engagement theory and employing a vigorous quasi-experimental design, our findings reveal that mindfulness training exerts a real impact on work-related outcomes, including employees’ experience of work meaningfulness, emotion regulation, a sense of job competence, and work engagement. In particular, work meaningfulness, emotion regulation, and job competence transmitted the effects of mindfulness training on employees’ engagement at work. These findings not only advance our understanding of the functioning of mindfulness theoretically, but also provide significant practical implications in organizational settings and beyond.

### Theoretical contributions

Our study advances the literature on mindfulness and work engagement in several notable ways. First, we provide a conceptual model connecting two research streams in the management field: mindfulness and work engagement. Although scholars have articulated the relationship between mindfulness and work engagement (e.g., Leroy et al., 2013; Malinowski and Lim, 2015), our empirical study goes one step further to explore how they are connected. Specifically, we follow Kahn’s theory of work engagement and articulate that mindfulness enhances work engagement because (a) mindfulness brings out employees’ awareness of their work meaningfulness; (b) mindfulness enhances emotion regulation; and (c) high mindful employees have high levels of attention and capabilities in learning new knowledge and skills and thus feel more confident in completing their tasks effectively. In doing so, our study answers the call to investigate work-specific outcomes of mindfulness (Dane, 2011) and the call to explore the mechanisms of mindfulness effects (Shapiro et al., 2006; Allen and Kiburz, 2012).

Second, our study contributes to the work engagement literature by introducing three essential psychological states conducive to work engagement. Previous research on work engagement has focused on personality characteristics (Langelaan et al., 2006), job demands/resources (Mauno et al., 2007), and personal states such as authentic functioning, positive affect, and psychological capital (Leroy et al., 2013; Malinowski and Lim, 2015). Extending this line of research, in this paper, we return to the cornerstone of engagement theory and refer to work meaningfulness, emotion regulation, and job competence to represent the three essential psychological states—meaningfulness, safety, and availability and investigate their central role in boosting employee engagement. Our findings therefore highlight the importance of Kahn’s theory in explaining the emergence of workplace engagement.

Furthermore, it is worthwhile to note that the three mediators influence work engagement differently. Arguably, mindfulness influences work meaningfulness and emotion regulation more directly than job competence. Regarding work meaningfulness, the decentering effects of mindfulness enable individuals to restore attention energies and broaden attention breath (Good et al., 2016), which enhance their awareness of the bigger picture of the work context, and ultimately the likelihood of realizing self-transcendence by perceiving the significance of their jobs (Breen, 2007). In terms of emotion regulation, one direct effect of mindfulness is to pause individuals’ automatic reactions to emotion stimuli (Good et al., 2016). Furthermore, mindfulness helps individuals get aware of and accept their emotions, therefore negative emotions (such as fear and anger) are effectively regulated (Gratz and Roemer, 2004). The mediating role of job competence is a result of attention control. Mindfulness allows individuals to reduce mind wandering and to focus on the work tasks with receptive awareness, which increase individual task proficiency (Brown et al., 2007). Employees are then likely to perceive their job competence. However, attention control consumes attentional resources which cannot last for a long time. Therefore, this can be one possible reason why the mediating role of job competence is not as strong as work meaningfulness and emotion regulation.

Moreover, the positive relationship between mindfulness intervention and emotion regulation is also supported by the studies on brain mechanism of mindfulness. Neuroimaging studies show that, during mindfulness practices, the brain regions involved in attention control mainly cover anterior cingulate cortex (ACC), medial prefrontal cortex (mPFC), and the striatum/basal ganglia (Petersen and Posner, 2012). At the same time, literature on the neuroscience of emotion regulation implicates the activation of brain systems for emotion regulation including ACC, insular cortex, and mPFC, and the deactivation of the amygdala (Posner et al., 2007, Wheeler et al., 2017). Thus, the overlap of brain regions associated with attention control and emotion-regulation suggests a neurobiological pathway whereby mindfulness meditation can exert its influences on at least the ACC and mPFC (Tang and Tang, 2015), indicating a positive association between mindfulness practices and emotion regulation.

Last but not least, our findings on the significant benefits of mindfulness in advancing individual job competence, emotion regulation, and work meaningfulness reveal mindfulness as an essential tool for employees to learn to utilize for personal improvement. Different from previous studies such as Leroy et al. (2013) that explore trait mindfulness as an individual difference, our findings demonstrate that state mindfulness can be trained to boost employee engagement. Thus in this paper, we suggest a feasible way (i.e., mindfulness training) for employees to get fully devoted at work, and thus enrich our understanding of approaches to improve employee work engagement.

### Practical implications

Given that work engagement is crucial for organizational survival and competitiveness in a rapidly changing business environment, its importance cannot be overemphasized (Rich et al., 2010). The findings from this study provide a clear roadmap to promote employee work engagement in the workplace. Our first implication is that companies are encouraged to offer mindfulness training for employees. In addition to helping employees reduce stress, such training can also enhance employee work engagement, thus helping them achieve high productivity and better at tackling problems encountered in work assignments.

Moreover, given the benefits of mindfulness training on developing individual job competence, emotion regulation, and perceived work meaningfulness, it is worthwhile for employees to persist in mindfulness practices on a daily basis. During our mindfulness training, in addition to class time, we also asked participants to practice the Mindfulness-Based Stress Reduction (MBSR) techniques for at least 20 min every day. Even if organizations do not provide mindfulness training opportunities, employees can learn relevant skills outside of the organization (such as meditations offered by a gymnasium or MBA classes), and, more importantly, practice such skills frequently to improve their experience of work meaningfulness, emotion regulation, and job competence in order to become physically, cognitively, and emotionally immersed at work.

### Limitations and future research directions

Despite our contributions, our study also has some limitations that need to be addressed in future research. First, we acknowledge that our quasi-experimental study design cannot claim causality for our proposed relationships. In our study, we used samples from existing MBA classes (mindfulness vs. non-mindfulness class) rather than randomly assign participants into experimental and control groups. In order to alleviate potential confounding effects due to such research design, we controlled for job demands and job resources to exclude potential impacts of job and task contexts on work engagement. In this vein, we suggest that future research conduct a laboratory study to examine if our findings can be replicated. Moreover, it is possible that individuals with high suggestibility are likely to be influenced by mediation practices (Gudjonsson, 1990; Mclachlan and Douglas, 2011), future experiments are therefore encouraged to control for respondents’ suggestibility levels.

Second, the fact that all our variables were self-reported by employees may raise concerns with common method variance (Podsakoff et al., 2012). To mitigate common method variance, we took two remedies. First, we created a separation in time (i.e., at six time points) between assessments of our predictor and criterion variables (Podsakoff et al., 2012). Second, we conducted a Harman’s single-factor test, and found that the first principal component accounted for only 18.38% of the total variance, which was far below the threshold of 50%, revealing that common method variance is less likely to be a concern in this study. Yet, we encourage future studies to test our variables from multiple sources.

Third, in this study, we did not explore boundary conditions for the effects of mindfulness on employee work engagement. Future studies can investigate if individual differences such as personality (John and Gross, 2007) and contextual factors such as job autonomy (e.g., Liu et al., 2011) affect the relationship between mindfulness and employee work engagement. For instance, individual neuroticism may reduce the effect of mindfulness on emotion regulation (e.g., John and Gross, 2007), thus weakening one’s emotional engagement at work. Finally, future research can explore the effects of these contingencies not only on the mindfulness-engagement relationship, but also extend our model to other organizational outcomes such as employee absenteeism and employee creativity.

## Conclusion

Based on Kahn (1990)’s grounded theory of work engagement, we propose that mindfulness is closely related to employee engagement through three psychological conditions—work meaningfulness, emotion regulation, and job competence. Our results from a quasi-experiment provided strong support for our theoretical model. Our findings advance our understanding regarding how and why state mindfulness benefits work engagement, as well as provide significant implications for management practices. We hope that our endeavor will inspire more studies to explore the impacts of mindfulness training on employee workplace behaviors and to look more closely into the pathways and contingencies for such impacts.

## Data availability statement

The data of this article will be available from the corresponding author upon reasonable request.

## Ethics statement

The studies involving human participants were reviewed and approved by the Research Ethics Committee in School of Business, East China University of Science and Technology. The patients/participants provided their written informed consent to participate in this study.

## Author contributions

LC and XL worked on the research design and theoretical building. LX worked on the data analysis and writing. All authors contributed to the article and approved the submitted version.
